# Molecular Mechanism for the Dual Alcohol Modulation of Cys-loop Receptors

**DOI:** 10.1371/journal.pcbi.1002710

**Published:** 2012-10-04

**Authors:** Samuel Murail, Rebecca J. Howard, Torben Broemstrup, Edward J. Bertaccini, R. Adron Harris, James R. Trudell, Erik Lindahl

**Affiliations:** 1 Science for Life Laboratory, KTH Royal Institute of Technology & Stockholm University, Stockholm, Sweden; 2 Center for Biomembrane Research, Department of Biochemistry & Biophysics, Stockholm University, Stockholm, Sweden; 3 Waggoner Center for Alcohol and Addiction Research, The University of Texas at Austin, Austin, Texas, United States of America; 4 Department of Anesthesia, Palo Alto Veterans Affairs Health Care System, Palo Alto, California, United States of America; 5 Department of Anesthesia and Beckman Program for Molecular and Genetic Medicine, Stanford University School of Medicine, Stanford, United States of America; University of Illinois, United States of America

## Abstract

Cys-loop receptors constitute a superfamily of pentameric ligand-gated ion channels (pLGICs), including receptors for acetylcholine, serotonin, glycine and γ-aminobutyric acid. Several bacterial homologues have been identified that are excellent models for understanding allosteric binding of alcohols and anesthetics in human Cys-loop receptors. Recently, we showed that a single point mutation on a prokaryotic homologue (GLIC) could transform it from a channel weakly potentiated by ethanol into a highly ethanol-sensitive channel. Here, we have employed molecular simulations to study ethanol binding to GLIC, and to elucidate the role of the ethanol-enhancing mutation in GLIC modulation. By performing 1-µs simulations with and without ethanol on wild-type and mutated GLIC, we observed spontaneous binding in both intra-subunit and inter-subunit transmembrane cavities. In contrast to the glycine receptor GlyR, in which we previously observed ethanol binding primarily in an inter-subunit cavity, ethanol primarily occupied an intra-subunit cavity in wild-type GLIC. However, the highly ethanol-sensitive GLIC mutation significantly enhanced ethanol binding in the inter-subunit cavity. These results demonstrate dramatic effects of the F(14′)A mutation on the distribution of ligands, and are consistent with a two-site model of pLGIC inhibition and potentiation.

## Author Summary

Communication from one nerve cell to the next is an essential process for brain and muscle function. Nerve impulses result in release of transmitter molecules from one cell that bind to receptors on the next cell. Transmitter binding opens a pore in each receptor and ions flow across the membrane, leading to either enhancement or inhibition of new nerve impulses. These receptors are modulated by numerous drugs, including alcohols and anesthetics; identifying the precise location of modulator binding is critical for drug development. We have used computer simulation methods to model alcohol diffusion and binding to a receptor. By modifying a single residue in the receptor, we were able to move the location of the binding site and dramatically alter alcohol modulation, which supports a model with two separate binding sites for enhancement and inhibition in this family of receptors.

## Introduction

Synaptic transmission is one of the most important functions of our nervous system, and modulation of post-synaptic receptors is of tremendous importance to understanding the effects of toxins, neuropharmaceuticals, drugs of abuse, and anesthetics, as well as the physiological basis for consciousness. Ethanol is likely the oldest drug known to man, and has been identified as a modulator of synaptic transmission. Ethanol affects the central nervous system by interacting with several proteins, in particular post-synaptic ion channel receptors. Among these, several key targets of alcohol modulation fall in the family of Cys-loop receptors (see reviews [1], [2]).

Cys-loop receptors constitute a family of pentameric ligand-gated ion channels (pLGICs). These receptors are activated by a variety of ligands, from which they draw their names: they include the cation-conducting nicotinic acetylcholine receptors (nAChRs) and serotonin-3 receptors, and the anion-conducting glycine receptors (GlyRs) and γ-aminobutyric acid-A receptors (GABA_A_Rs). In addition to activation by their respective ligands, pLGICs exhibit allosteric modulation by numerous endogenous and exogenous molecules, including alcohols and anesthetics. The dual action of these molecules on pLGICs is particularly interesting. Alcohols and anesthetics potentiate many anionic channels (GlyRs and most GABA_A_Rs [3]–[5]), whereas only short-chain alcohols potentiate nAChRs [6]; conversely, longer-chain alcohols and most anesthetics inhibit nAChRs [6], and both types of modulators inhibit the ρ subtype of GABA_A_Rs [7].

Despite their apparent functional diversity, pLGICs share an overall architectural organization, with five subunits and three distinct domains [8]. The extracellular domain (ECD) contains the agonist site, at which binding leads to opening of a central pore in the transmembrane domain (TMD). Each TMD contains four transmembrane helices (M1–M4), with the M2 helices lining the pore; residues in M2 are often described using prime notation, beginning ∼1′ at the N-terminal intracellular end and progressing to ∼20′ at the C-terminal extracellular end of the TMD. A third, intracellular loop domain (ILD) is present in some family members, where it modifies functional properties such as desensitization [8]. By definition, allosteric modulators alter the energy landscape for channel activation by binding at a location distinct from the primary ligand-binding site (see review [9]). Modulators including alcohols and anesthetics have been shown to regulate activation by binding at least partially in the TMD [10], [11]. In addition, at high concentrations some modulators and endogenous steroids can activate GABA_A_R by themselves [9], [12], [13]. Until recently, no high-resolution structures of the pLGIC TMD were available, and the lower-resolution structures [14] or models [15] of human receptors have not allowed definitive characterization of the allosteric binding site(s). Because pLGICs are pharmaceutical targets for large classes of molecules including cannabinoids, steroids, barbiturates, and general anesthetics [9], [16], identification of the binding sites and mechanisms of action of these molecules is critical to designing better drugs.

Our understanding of pLGIC structure has advanced tremendously in the last five years with the publication of the first crystallographic structures of three different receptors in this family. The first two structures, ELIC and GLIC, were of pLGICs from the prokaryotes *Erwinia chrysanthemi* [17] and *Gloeobacter violaceus* [18], [19], and have already provided valuable templates for homology models of human receptors such as GlyRs. We previously used a GlyR model based on GLIC to show spontaneous ethanol binding to a site between subunits [20], consistent with past studies based on lower-resolution pLGIC structures [21]. The third pLGIC to be crystallized, the GluCl channel from the eukaryote *Caenorhabditis elegans*, was co-crystallized with a partial allosteric agonist bound between subunits [22], again consistent with functional enhancement mediated by binding in this region. Conversely, the GLIC receptor was recently co-crystallized with the anesthetics propofol and desflurane [23] bound to an intra-subunit pocket in the upper part of the TMD. Resolving the contributions of inter- and intra-subunit binding is critical to understanding the structural basis for pLGIC allostery.

One explanation for the observation of both inter- and intra-subunit binding could be the contribution of multiple allosteric sites to different modulatory effects. Like other cationic pLGICs, GLIC is inhibited by most anesthetics [24] and long-chain alcohols, while it exhibits weak potentiation by methanol and ethanol [25]. We previously showed that the mutation F(14′)A transforms GLIC into a highly ethanol-sensitive channel that is potentiated by alcohols as large as hexanol [25], thus more closely approximating the properties of GlyRs and GABA_A_Rs [4], [5]. We further demonstrated by molecular dynamics that the enhanced potentiation of the F(14′)A variant correlated with expansion of the inter-subunit cavity [25]. Thus, inter-subunit ethanol binding may correspond to enhanced function of pLGICs including GlyRs [20] and the GLIC F(14′)A mutant [25], while the crystallographically determined intra-subunit binding of anesthetics on GLIC [23] could represent an independent inhibitory site of action.

To address this hypothesis and further elucidate the effects of the F(14′)A mutation in GLIC, we have systematically explored binding of ethanol to GLIC WT and F(14′)A receptors in molecular dynamics simulations. Four molecular systems were created to study both the WT and mutant, with and without ethanol present in the bulk solvent, and both binding and equilibrium exchange of ethanol in identified TMD cavities was quantified. We also quantified the F(14′)A mutant with a single ethanol molecule bound in each of the five inter-subunit cavities. In our simulations, ethanol bound in both sites but primarily occupied the intra-subunit cavity of WT GLIC, in contrast to our previous GlyR simulations conducted under identical conditions [20], but in agreement with the anesthetic co-crystal structures of GLIC [23]. The single point F(14′)A mutation was sufficient to enhance the average number of ethanol molecules observed in the inter-subunit more than twofold. Given our previous experimental results showing low sensitivity of WT and high sensitivity of F(14′)A to ethanol [25], these data support a two-site model for modulation of pLGICs, involving both an inhibiting intra-subunit site and an potentiating inter-subunit site of action.

## Results

### Localized effects of the F(14′)A mutation on the structure of GLIC

**Figure 1:**
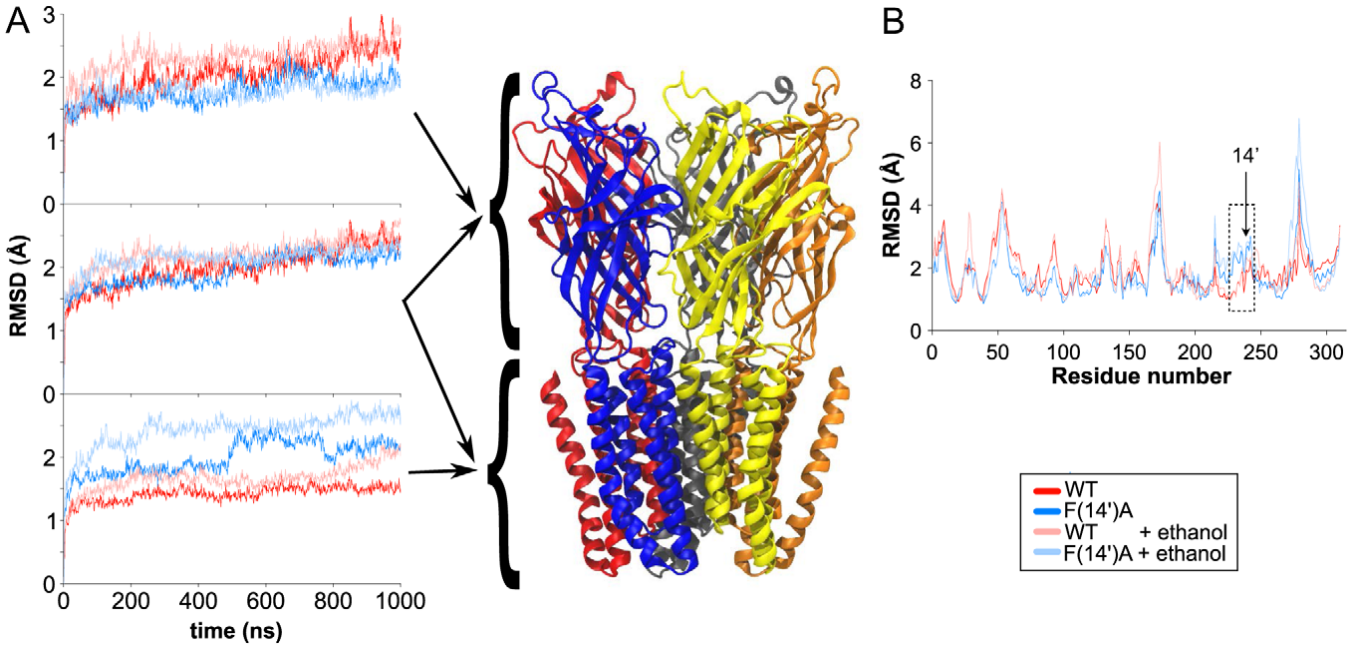
Structural deviation of GLIC simulations. (*A*) C_α_ RMSD of the ECD (*upper panel*), whole protein (*middle panel*), and TMD (*lower panel*) for the WT (dark red), F(14′)A (dark blue), WT+ethanol (light red), and F(14′)A+ethanol (light blue) simulations relative to the GLIC crystal structure (PDB ID 3EAM). Right hand panel shows the GLIC structure colored by chain. (*B*) Average RMSD per residue over the four 1-µs simulations, colored as in (A). ECD includes residues 5 to 195, TMD residues 196 to 315. Box indicates high-RMSD residues 222–245, with residue 238 labeled according to M2 prime notation (14′).

We performed 1-µs simulations of WT and F(14′)A GLIC in fully solvated lipid-embedded systems. The protonation state (pH 4.6) corresponding to the crystallization conditions of the template GLIC structure was identical to the one proposed by Bocquet *et al.* [18] and also used by other groups [26], [27]. Both the WT and F(14′)A simulations exhibited relatively small deviations from the GLIC crystal structure, with the overall protein C_α_ root mean square deviations (RMSD) under 3 Å in both cases. Indeed, over the last 100 ns, the C_α_ RMSD relative to the crystal structure was 2.43±0.12 Å for the WT and 2.18±0.08 Å for F(14′)A (figure 1A, middle panel), below the average X-ray resolution of the protein (2.90 Å).

Although the overall structures of the WT and F(14′)A channels were similar throughout the simulations, comparing the ECD and TMD of each protein revealed intriguing differences. In place of the extended ILD found in metazoan pLGICs, GLIC contains only a short linker that cannot be considered an independent domain [28]. The F(14′)A mutation, which is located in the TMD, was associated with TMD packing rearrangements that led to a larger local change of the structure: the average TMD C_α_ RMSD over the last 100 ns was 1.51±0.06 Å for the WT and 2.20±0.07 Å for the F(14′A) mutated system (figure 1A, lower panel). This increased TMD deviation was compensated in the overall RMSD by decreased structural fluctuations of the ECD: the ECD C_α_ RMSD over the last 100 ns was 2.51±0.18 Å for WT but 1.96±0.10 Å for F(14′)A (figure 1A, upper panel).

Calculating the average RMSD per residue (figure 1B) exposed selective deviation of the M2 helix (residues 222 to 245) in the mutated system, approximately 2.2 Å for F(14′)A versus 1.2 Å for the WT. A visual inspection of the trajectory revealed a kink in the M2 helix. This kink appeared quickly, after only a few nanoseconds of simulation. Whereas in the WT simulation the average kink angle (12.46±2.36°) remained close to the crystal structure value (8.01°), the average angle in the F(14′)A simulation stabilized around double the WT value (22.18±2.04°).

**Figure 2:**
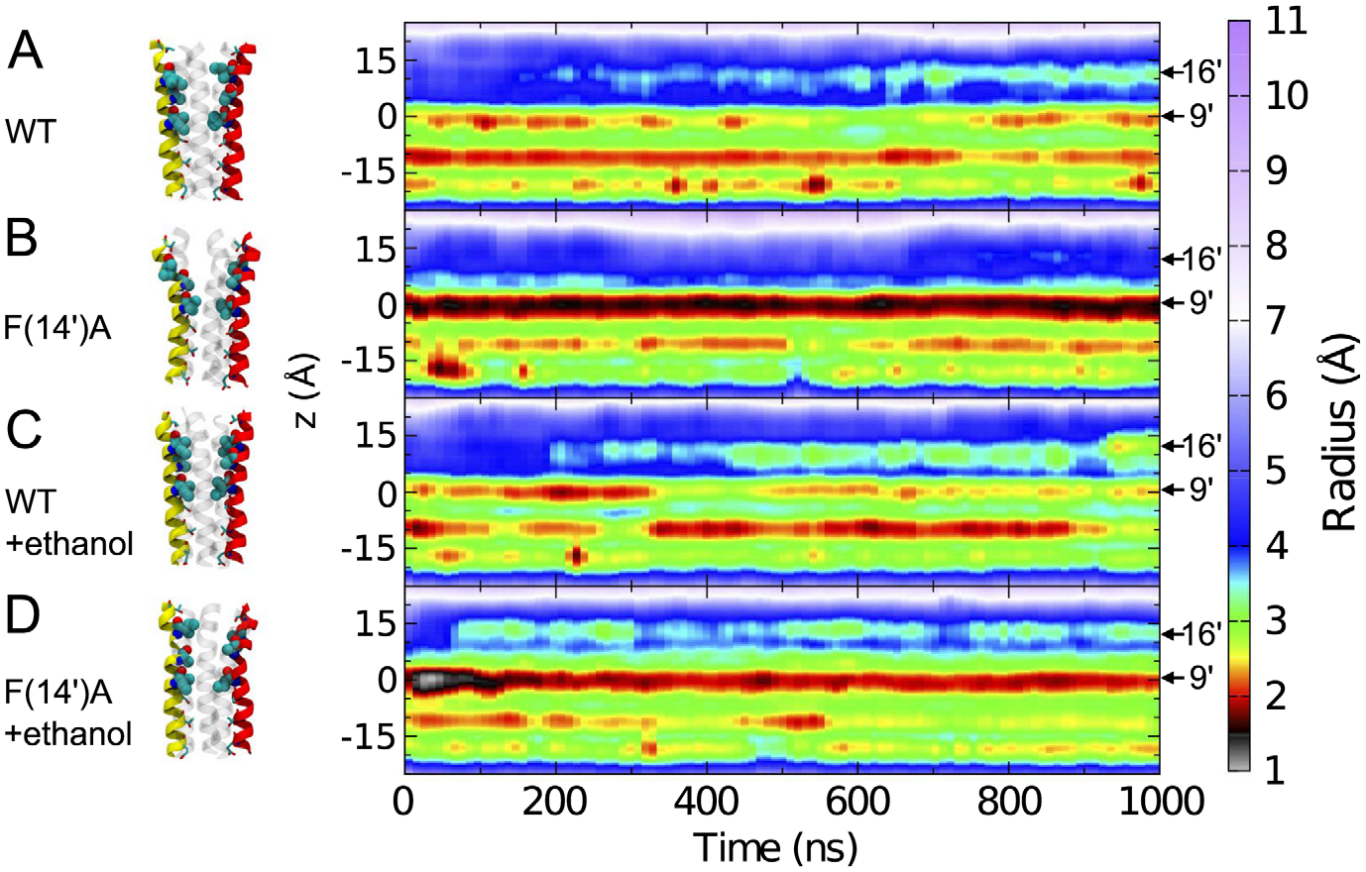
Pore constrictions. Pore radius as a function of membrane z-axis plane (left axis) and time (lower axis) for the (*A*) WT, (*B*) F(14′)A, (*C*) WT+ethanol and (*D*) F(14′)A+ethanol simulations. Prominent constriction points were observed around 10 Å/I(16′), 0 Å/I(9′), −10 Å/T(2′), and −18 Å/E(−2′). Structures on the left show the pore helices at the end of the 1-µs simulation with two M2 helices colored red and yellow and the three others as transparent cartoons. Residues lining the pore are shown as spheres for hydrophobic residues I(16′) and I(9′) and as sticks for polar residues T(20′), S(6′), T(2′) and E(−2′).

We observed further indirect effects of the F(14′)A mutation on the M2 structure via constriction of the channel pore. Monitoring the pore radius across the ∼30-Å TMD throughout the 1-µs WT simulation (figure 2A) revealed a pore constriction of radius 2.25±0.31 Å around residue I(9′), which was previously shown to comprise the GLIC hydrophobic permeation barrier [29]. Past studies showed a pore constriction of these dimensions to be wide enough to let some Cl^−^ ion pass through the GlyR pore [20]. Similarly, PMF studies of GLIC have shown that at the same level radii of ∼2.4 Å [30] or ∼2.5 Å [31] were compatible with a conducting channel. However, the F(14′)A mutation tightened the pore constriction at the I(9′) position (figure 2B) to an average radius of 1.60±0.22 Å. Thus, WT GLIC appeared to be completely open, whereas we presumed the F(14′)A mutant to be mainly closed. This finding is consistent with our previous observation that the F(14′)A mutation shifts gating over 0.5 pH units to the right, corresponding to an approximately four-fold decrease in proton sensitivity [25]. Although the nonconducting F(14′)A model was structurally distinct from other recent closed [17] or locally-closed [32] pLGIC models, the relevance and relative contributions of these and other possible nonconducting conformations to GLIC gating remain to be determined. Moreover, our F(14′)A model might only reflect the increased flexibility of M2 upper part, rather than a new GLIC conformation.

### F(14′)A effects on cavity volume

**Figure 3:**
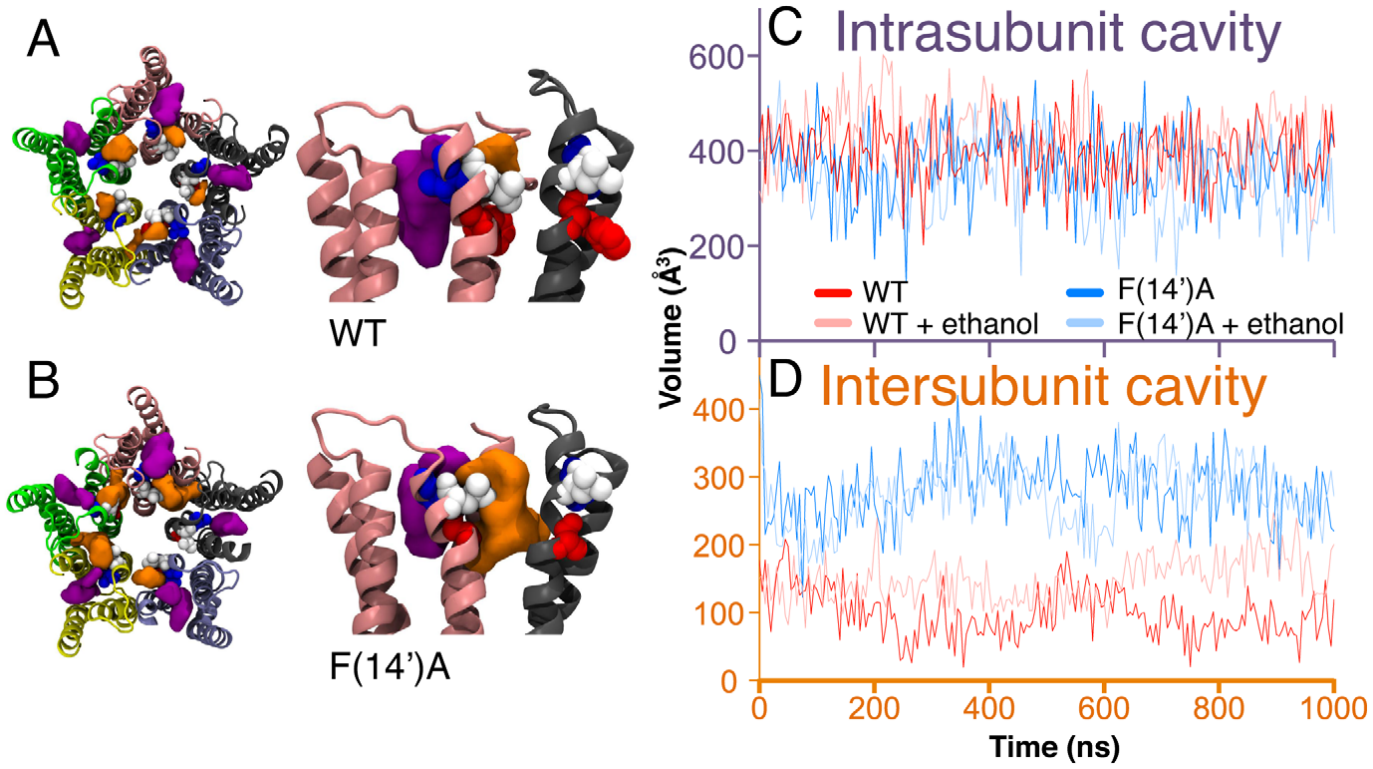
Effects of the F(14′)A mutation on the cavity volume. Structures of the WT (*A*) and F(14′)A (*B*) TMDs superposed with their intra-subunit and inter-subunit cavities averaged over 1 µs. Views from the extracellular side (*left*) and membrane plane (*right*) display the TMD colored by subunit. Positions 14′, 17′, and 18′ are represented as red, white, and blue spheres, respectively. The average intra- and inter-subunit cavities are represented by violet and orange surfaces. (*C*) Intra-subunit and (*D*) inter-subunit cavity volumes averaged across five subunits in the WT (dark red), F(14′)A (dark blue), WT+ethanol (light red), and F(14′)A+ethanol (light blue) simulations.

In both WT and F(14′)A simulations, we identified two major TMD cavities for each of the five protein subunits. The biggest cavities were intra-subunit, and were located towards the extra-cellular side of the TMD, facing the membrane (figure 3A–B, violet). These cavities were hydrophobic, as confirmed by their negligible hydration and their occupancy by lipid fatty acid chain atoms (table 1): average lipid occupancy over the second half of the simulation measured 5.8±0.9 and 6.3±0.9 atoms per cavity for the WT and F(14′)A trajectories, respectively. Accordingly, the intra-subunit cavities were mainly lined by hydrophobic residues, with only 20% polar accessible surface area in WT and 22% in F(14′)A simulations. These cavities did not exhibit systematic changes in volume during the simulations, and did not appear to be influenced by the F(14′)A mutation, with average volumes of 368±68 Å^3^ for WT and 392±64 Å^3^ for F(14′)A (figure 3C).

**Table 1:** Intra-subunit cavity properties.

		1	2	3	4	5	Average
**Volume** a	**WT**	549±139	304±128	418±193	228±147	340±160	368±68
	**F(14′)A**	601±141	386±186	342±159	407±134	223±137	392±64
	**WT+ethanol**	500±155	569±126	139±154	502±159	321±185	406±64
	**F(14′)A+ethanol**	450±169	235±177	339±178	346±200	188±147	312±72
**Lipid** b	**WT**	9.9±2.7	3.3±1.6	7.3±2.6	3.2±1.2	5.5±2.0	5.8±0.9
	**F(14′)A**	9.7±2.8	6.7±2.4	4.9±1.7	6.8±2.0	3.5±1.8	6.3±0.9
	**WT+ethanol**	4.4±3.5	4.0±2.8	1.7±1.9	8.7±2.7	2.9±2.9	4.3±1.0
	**F(14′)A+ethanol**	7.0±3.1	0.8±1.5	3.2±2.1	4.9±1.4	0.7±1.2	3.3±1.0
**Water** c	**WT**	0.1±0.4	0.7±0.8	0.1±0.4	0.1±0.2	0.0±0.2	0.2±0.2
	**F(14′)A**	0.1±0.5	0.0±0.2	0.1±0.3	0.2±0.5	0.1±0.3	0.1±0.2
	**WT+ethanol**	0.3±0.7	0.4±0.7	0.1±0.4	0.1±0.4	0.3±0.6	0.3±0.2
	**F(14′)A+ethanol**	0.2±0.6	0.1±0.2	0.1±0.3	0.0±0.1	0.1±0.2	0.1±0.1
**Ethanol** c	**WT+ethanol**	1.0±0.8	2.1±0.8	0.3±0.6	0.6±0.5	0.7±0.7	0.9±0.3
	**F(14′)A+ethanol**	0.7±0.5	1.2±0.7	0.7±0.7	0.0±0.2	0.9±0.5	0.7±0.2

Average volume and number of solvent molecules in each intra-subunit cavity, as well as cavity averages, computed over the second half (500 ns) of each simulation. Errors are standard deviations.

a Volume computed using mdpocket module of the Fpocket package, expressed in Å^3^.

b Average number of fatty acid side chain atoms.

c Average number of molecules.

In our previous work [25], we adopted the terminology of Nury et al. [23] identifying two interconnected cavities at each GLIC subunit interface: an “inter-subunit cavity” facing the membrane, and a “linking tunnel” facing the pore. However, these cavities were not consistently defined in our 1-µs simulations; in F(14′)A, they were generally indistinguishable. Therefore, in this work we defined a single inter-subunit cavity associated with each subunit interface, partially exposed to both the membrane and the pore.

The inter-subunit cavities were located in roughly the same plane as the intra-subunit cavities relative to the lipid bilayer, but were more hydrophilic, with 41% polar accessible surface in both WT and F(14′)A simulations, and occupancy by several water molecules in both simulations as well as the previously published crystal structures [18], [19]. No lipid occupancy was observed in the inter-subunit cavities. We noted that several of the charged or polar residues lining the inter-subunit cavities (N200, H235, N239, E243, K248, Y263) are conserved in human pLGICs, supporting the functional relevance of these cavities to gating, modulation, or assembly.

In contrast to the intra-subunit cavities, the inter-subunit cavities were dramatically altered by the F(14′)A mutation. In WT GLIC, each inter-subunit cavity was lined by residues in upmost turn of M2, including the MTS-accessible residues L(17′) and V(18′) [25], and the M2–M3 loop, and did not penetrate to the level of F(14′) (figure 3A). The average WT inter-subunit cavity occupied 96±33 Å^3^ during the second half of the simulation, less than a third of the volume of the average intra-subunit cavity, and was occupied by 1.0±0.4 water molecules (table 2). Conversely, the absence of the phenyl group in the F(14′)A mutant allowed the inter-subunit cavities to extend deeper towards the intracellular side, in some cases contacting the substituted alanines at 14′ (figure 3B). Accordingly, the average inter-subunit cavity volume was enlarged from the beginning of the simulation; by the second half, it increased to 283±45 Å^3^ (figure 3D), a threefold increase over WT (table 2). Furthermore, the increased volume allowed occupation by 4.4±0.8 water molecules, fourfold more than WT (table 2).

**Table 2:** Inter-subunit cavity properties.

		1	2	3	4	5	Average
**Volume** a	**WT**	138±95	62±52	181±80	64±64	36±38	96±33
	**F(14′)A**	178±159	496±134	136±86	23±32	580±154	283±45
	**WT+ethanol**	212±122	125±58	88±55	181±46	194±79	160±35
	**F(14′)A+ethanol**	180±60	559±112	293±77	73±78	267±150	274±45
**Water** b	**WT**	2.0±1.3	0.7±0.7	1.2±0.9	0.7±0.9	0.6±0.8	1.0±0.4
	**F(14′)A**	2.6±2.8	7.8±1.9	1.5±1.1	1.5±0.9	8.7±2.4	4.4±0.8
	**WT+ethanol**	2.1±1.5	0.3±0.5	0.8±1.0	0.3±0.7	1.1±0.9	0.9±0.4
	**F(14′)A+ethanol**	1.9±1.0	4.5±1.9	1.3±0.9	0.8±0.5	1.5±1.3	2.0±0.5
**Ethanol** b	**WT+ethanol**	0.3±0.5	0.2±0.4	0.0±0.0	0.8±0.4	0.4±0.5	0.3±0.2
	**F(14′)A+ethanol**	0.6±0.5	1.6±0.7	1.2±0.4	0.0±0.0	0.7±0.7	0.8±0.2

Average volume and number of solvent molecules in each inter-subunit cavity, as well as cavity averages, computed over the second half (500 ns) of each simulation. Errors are standard deviations.

a Volume computed using mdpocket module of the Fpocket package, expressed in Å^3^.

b Average number of molecules computed.

### Localized effects of ethanol on GLIC structure

To identify sites and consequences of ethanol binding on GLIC, and the effect of the F(14′)A mutation on ethanol interactions, we ran additional molecular dynamics simulations of both WT and F(14′)A in the presence of ethanol. We placed each of the previously defined systems in ∼600 mM ethanol by replacing 1% of the bulk water molecules with ethanol. We previously showed that a similar concentration, approximately 3 times the concentration associated with immobilization of organisms [1], potentiated GLIC WT weakly and F(14′)A potently [25]. After equilibration, we simulated both systems for 1 µs.

Ethanol had a limited effect on GLIC structure, increasing structural deviations in the TMD of both WT and F(14′)A. For WT, this increase was reflected in an average C_α_ RMSD with ethanol of 2.02±0.11 Å over the last 100 ns—a 34% increase over the ethanol-free simulation (figure 1A, lower panel). Similarly, the average C_α_ RMSD for the F(14′)A TMD with ethanol was 2.68±0.08 Å over the last 100 ns, a 22% increase (figure 1A, lower panel). However, structural deviations averaged over the whole protein (figure 1A, middle panel) or the ECD (figure 1A, upper panel) were similar with and without ethanol for both WT and F(14′)A. The average RMSD per residue (figure 1B), M2 kink angle (respectively, 11.02±1.81° and 24.25±2.25° for WT and F(14′)A GLIC *versus* 12.46±2.36° and 22.18±2.04° without ethanol) and intra-subunit cavity volumes (figure 3C) also followed similar patterns with and without ethanol for each system.

Whereas ethanol had little effect on the WT pore radius (figure 2C), it partially compensated for the constricted pore in F(14′)A (figure 2D). The F(14′)A pore radius at the level of the I(9′) barrier stabilized around 2 Å in the presence of ethanol (figure 2D), ∼25% larger than in the ethanol-free simulation (figure 2B), and only ∼11% smaller than in the WT simulations (figures 2A,C). Conversely, ethanol selectively increased the average inter-subunit cavity volume in the WT simulation (figure 3D) from 96±33 Å^3^ to 160±35 Å^3^ (table 2). The equivalent cavities in F(14′)A occupied 283±45 Å^3^ and 274±45 Å^3^ (table 2), consistently larger than in WT, but unaltered by ethanol (figure 3D).

### Differential ethanol binding in GLIC TMD cavities

**Figure 4:**
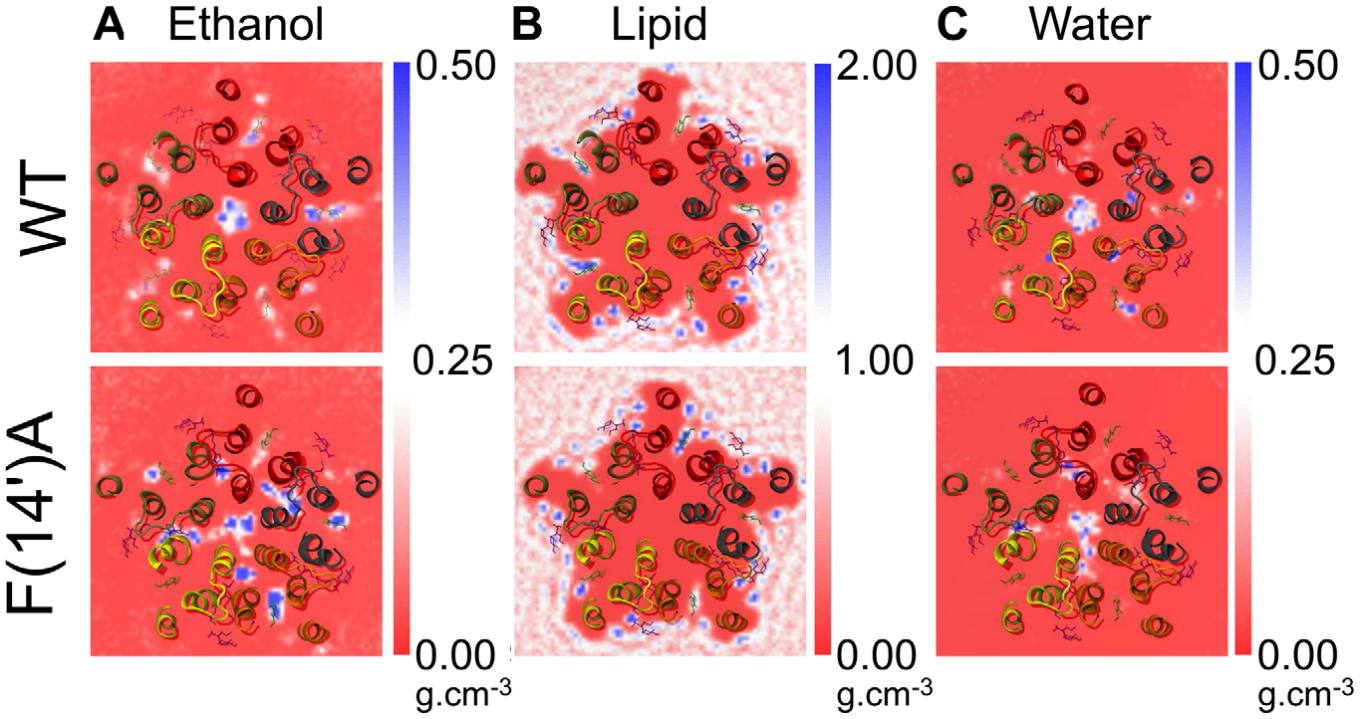
Effect of F(14′)A on the occupancy. (*A*) Ethanol, (*B*) DOPC, and (*C*) water density in the TMD. Densities were averaged over the complete trajectory of the WT (*upper panels*) and F(14′)A (*lower panels*) simulations with ethanol. The TMD is colored by chain. For comparison, crystal structures of the GLIC complex with propofol (PDB ID 3P50) [23] and the GluCl complex with ivermectin (PDB ID 3RHW) [22] were aligned on the TMD C_α_ atoms, and the corresponding propofol and ivermectin molecules were displayed as green and pink sticks, respectively. For WT, ethanol bound predominantly in the intra-subunit cavity in a similar position to propofol; in F(14′)A, ethanol bound to the intra- and inter-subunit cavities equivalently.

Our ethanol simulations allowed us to directly observe ethanol occupation of both the intra-subunit and inter-subunit cavities. During the WT simulation, ethanol primarily occupied the intra-subunit cavities (figure 4A, upper panel). An average of 0.9±0.3 and 0.3±0.2 ethanol molecules were present in each intra and inter-subunit cavity, respectively, over the second half of the simulation. In contrast, the F(14′)A mutation increased ethanol occupancy in the inter-subunit cavities almost threefold, approximating the occupancy of the intra-subunit cavities, which was similar to WT (figure 4A). Average occupancies in the F(14′)A simulation were 0.7±0.2 (table 1) and 0.8±0.2 (table 2) for the intra- and inter-subunit cavities, respectively. Ethanol occupation of the F(14′)A inter-subunit cavities also exhibited substantial variability: for example, one of the inter-subunit cavities was occupied by an average of ∼2 ethanol molecules throughout the second half of the simulation, while another failed to bind ethanol (table 2).

**Figure 5:**
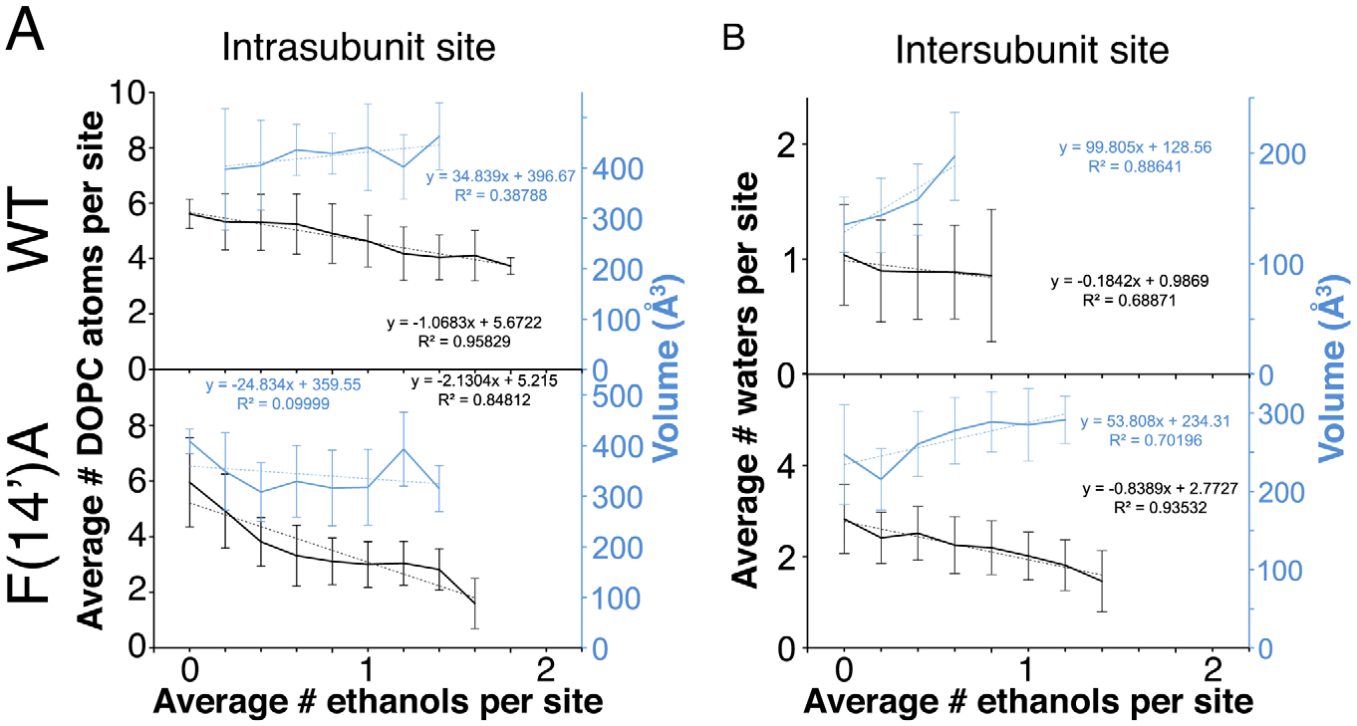
Cavity occupancy correlations. (*A*) Average number of DOPC lipid atoms (black, left axis) and average cavity volume (blue, right axis) for each intra-subunit cavity as a function of the average number of ethanol molecules in the same site, with standard errors. (*B*) Average number of water molecules (black, left axis) and average cavity volume (blue, right axis) for each inter-subunit cavity as functions of the average number of ethanol molecules in the same site, with standard errors. In (*A*) and (*B*), linear fits (dotted lines) are also shown, with the corresponding equations and correlation values (R^2^).

In the intra-subunit cavities, ethanol bound between the M1 and M3 helices of each subunit in a pose similar to that of propofol in the recent co-crystal structure [23] (figure 4A). Ethanol binding corresponded to decreased lipid occupancy in the same cavities (figure 4B): average intra-subunit lipid fatty acid chain atoms decreased from 5.8±0.9 (WT) and 6.3±0.9 (F(14′)A) without ethanol to 4.3±1.0 (WT) and 3.3±1.0 (F(14′)A) with ethanol (table 1). As shown in figure 5A, we observed a negative correlation between the average number of ethanol molecules and lipid atoms occupying each intra-subunit cavity at a given time in the WT (R^2^ = 0.96) and F(14′)A (R^2^ = 0.85) simulations. Conversely, there was no correlation between the average number of ethanol molecules at a given time and the average volume of the intra-subunit cavities (figure 5A). Thus, ethanol binding in the intra-subunit cavities displaced lipid binding without altering cavity volume.

Whereas WT ethanol binding was difficult to observe in the inter-subunit cavities, being occupied less than one-third of the time, ethanol clearly bound in the F(14′)A inter-subunit cavities near the M2 helices and the channel pore (figure 4A, lower panel). Water occupied some of the same cavities (Figure 4C), and we observed a negative correlation between the average number of ethanol and water molecules occupying each inter-subunit cavity at a given time in both the WT (R^2^ = 0.69) and F(14′)A (R^2^ = 0.94) simulations (figure 5B). There was also a positive correlation between inter-subunit ethanol occupancy and cavity volume in both the WT (R^2^ = 0.89) and F(14′)A (R^2^ = 0.70) simulations (figure 5B). Thus, ethanol binding in the inter-subunit cavities may have dual effects of displacing water and increasing cavity volume.

**Figure 6:**
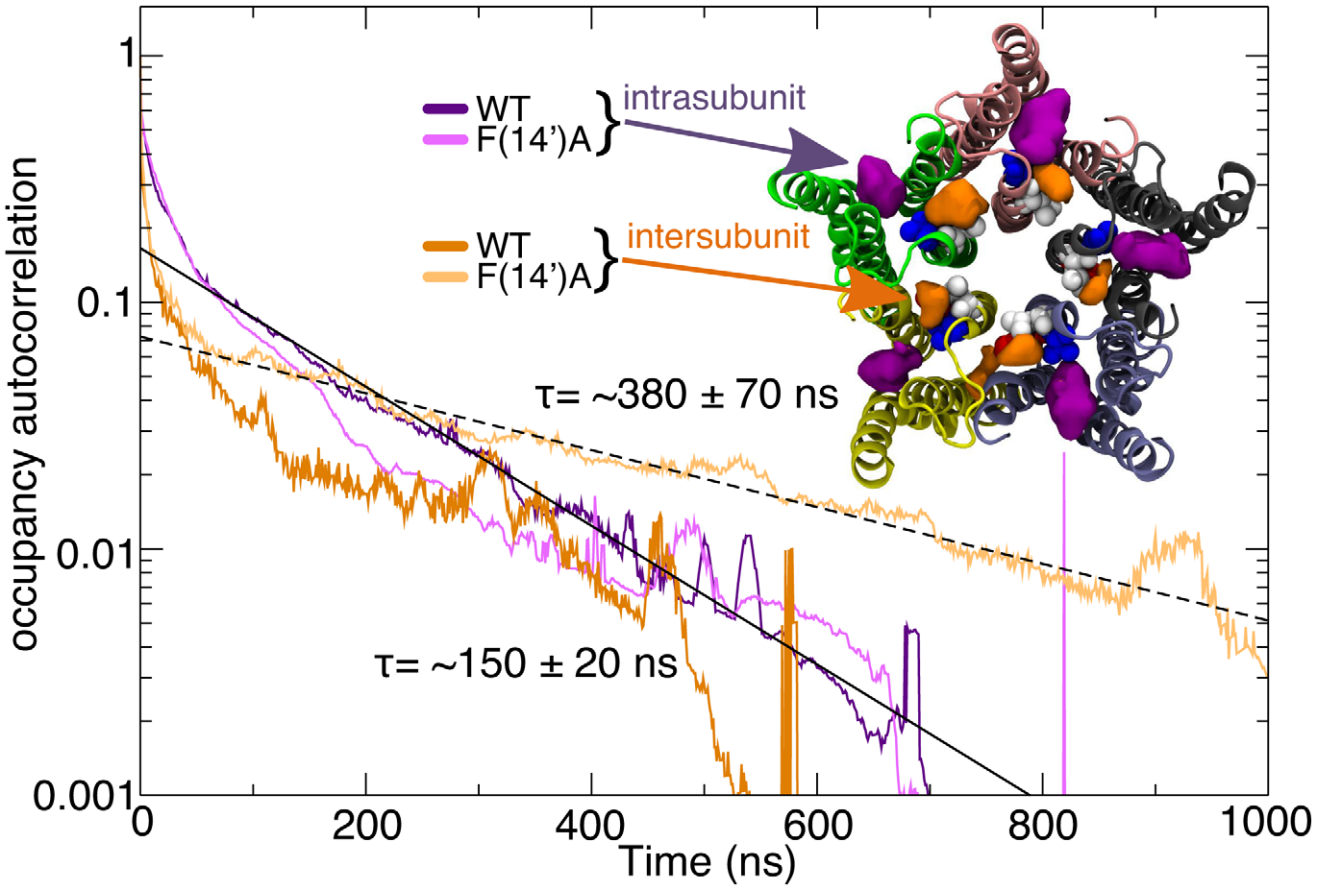
Ethanol exchange rates. Occupancy autocorrelations for ethanol molecules that exhibit cavity occupancy at some point in each simulation, colored according to the site and system. The slow component of ethanol exchange was fit by an exponential (solid line) with τ∼150 ns and a standard error of 20 ns for the intra-subunit WT (dark purple). The F(14′)A (light purple) site is not does not exhibit any statistically significant difference, and the very limited amount of ethanols present inter-subunit for WT (dark orange) makes a fit difficult. In contrast, the inter-subunit F(14′)A cavity (light orange) exhibits significantly slower exchange, fit by an exponential (dashed) with τ∼380 ns and a standard error of 70 ns.

Enhanced inter-subunit binding in the F(14′)A simulation also manifested in a slower exchange time between bound and bulk ethanol. As shown in figure 6, ethanol exchange in each cavity type was fit by a double-exponential model. The fast component (roughly 20 ns) of the exchange likely corresponds to molecules repeatedly moving in/out of cavities before or after binding. For the WT, ethanol present in the inter-subunit site has an exchange time constant (τ) of ∼150±20 ns, while the ethanol located in the inter-subunit cavity of the F(14′)A mutant has a considerably slower exchange, τ∼380±70 ns. While these point to significant relative differences, the values are not trivial to compare to experiments since they are sensitive to the cavity definition, simulation relaxation, and not least that they don't account for molecules re-entering the cavity before reaching bulk water.

**Figure 7:**
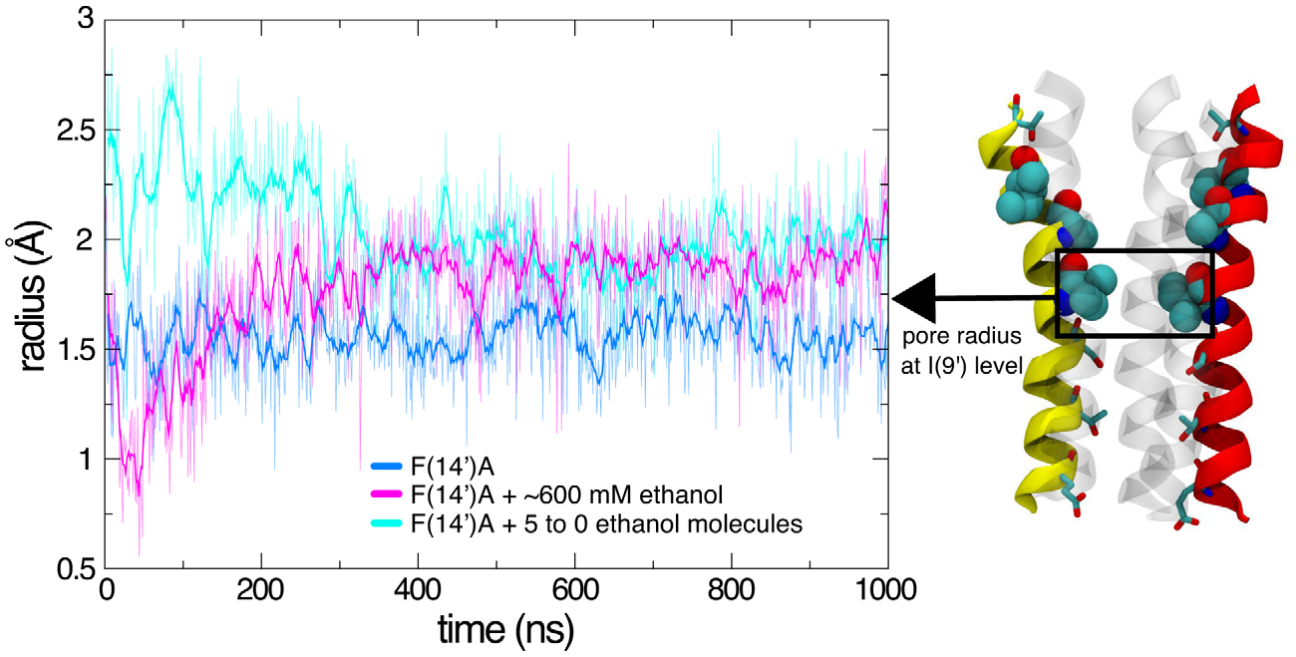
Effects of spontaneous and constrained ethanol binding on pore constriction. Minimal pore radius at the hydrophobic barrier at I(9′) as a function of time for F(14′)A simulations without ethanol (blue), with 600 mM ethanol (purple), and with one ethanol molecule constrained in each of the five inter-subunit cavities (cyan), computed by extracting the smallest pore radius around I(9)′ with a z-window of 5 Å. In the constrained system, one ethanol molecule was removed every 100 ns between 500 and 1000 ns.

To further investigate the effects of inter-subunit ethanol binding on F(14′)A structure, we performed an additional molecular dynamics simulation on the mutant with constrained ethanol molecules. Beginning with the ethanol-free system, we inserted one ethanol molecule in each of the five inter-subunit sites of F(14′)A, then simulated the system for 500 ns with the ethanol molecules constrained in the cavities. We then continued the simulation for another 500 ns, removing one ethanol molecule every 100 ns. As shown in figure 7, the F(14′)A pore radius at the level of I(9′) stabilized under these conditions around 2 Å, similar to the 600 mM-ethanol simulation and ∼25% larger than in the ethanol-free simulation. Early time points in the simulation trajectories showed even larger deviations: during the first 300 ns, the minimal pore radius at I(9′) in the constrained F(14′)A system oscillated between 2.0 and 2.5 Å, similar to the presumed-open WT system, before stabilizing around 2 Å between 300 and 500 ns. This enlarged pore radius relative to the ethanol-free system was stable upon sequential removal of the constrained ethanol molecules (figure 7). Conversely, in the presence of 600 mM ethanol, the I(9′) barrier had become extremely narrow at the beginning of the simulation, with the pore radius falling to as little as 1 Å. Visual inspection of the trajectory showed one of the subunits transiently moving towards the pore and partially occluding it. Following this initial constriction, the radius at the level of I(9′) progressively increased, stabilizing at 150 ns around 2 Å (figure 7). Thus, both constrained and spontaneous ethanol binding resulted in initial fluctuations of the pore radius at the I(9′) constriction point, but subsequently stabilized to similarly expanded dimensions, an effect which was not reversible over a 500-ns time scale.

## Discussion

As we previously reported [25], the mutation F(14′)A reduced agonist sensitivity and dramatically enhanced ethanol potentiation in the prokaryotic receptor GLIC. We sought to elucidate the structural basis for these functional effects by extending our previous 200-ns simulations of WT and F(14′)A GLIC in a fully solvated, lipid bilayer-embedded system [25] up to 1 µs. Furthermore, we took advantage of this system to simulate differential effects of ethanol binding on the closely related WT and F(14′)A receptors. Our results support a two-site model for allosteric modulation of pLGICs.

### Validation of molecular dynamics approach

During all simulations, the backbone structures of GLIC WT and F(14′)A were relatively stable, with total C_α_ RMSD under 3 Å. Deviations associated with the F(14′)A substitution and/or with ethanol solvation were localized to discrete regions of the protein, particularly the M2 helix. The structural integrity of our GLIC models relative to the crystallographic template supported the validity of our simulation conditions. In particular, we chose a physiologically extreme concentration of ethanol for our binding simulations to compensate for the low potency of ethanol for WT GLIC *in vitro* and to increase our sampling of low-occupancy binding sites within our 1-µs simulations. By replacing 1% of water molecules with ethanol, we approximated a 1 mol-% or ∼600 mM ethanol concentration, approximately 3 times the immobilizing concentration and over 30 times the legal blood alcohol concentration limit to drive a car in the United States [1]. Nonetheless, neither the WT nor F(14′)A models were systematically disrupted by this high concentration of ethanol; instead, they stabilized on a time scale comparable with the ethanol-free simulations.

Our simulations also confirmed the binding of various agents predicted from recent crystal structures. As observed in the earliest GLIC structures [18], [19], the membrane-facing intra-subunit cavities in our WT simulations were occupied by lipid, while the more hydrophilic inter-subunit cavities contained water. Furthermore, the ethanol-solvated WT simulation revealed intra-subunit ethanol binding that overlapped with the crystallographic propofol-binding site [23], consistent with the similar effects of propofol and ethanol on some pLGICs [4].

Despite the overall consistency of our simulations, the F(14′)A mutation did have structural consequences beyond the absence of the phenylalanine side chain. For example, the mutation increased RMSD through most of the M2 helix, systematically increased the M2 helix kink angle, and constricted the pore radius at the level of the I(9′) hydrophobic gate. These structural consequences highlight the indirect effects of point mutations that may dramatically alter functional properties, and underscore the value of molecular dynamics simulations in interpreting mutagenesis data. Furthermore, high variability of the M2 region relative to the rest of the protein is consistent with the non-periodic accessibility of mutated M2 residues reported by Parikh and coworkers [33], and could reflect increased mobility of this region under mutated or otherwise noncrystallographic conditions. The M2 helix may comprise a mobile structural element in which point mutations or the binding of allosteric modulators could influence the equilibrium constant of pore gating transitions.

As in our previous work [25], we imposed an acidic protonation state (pH 4.6) corresponding to the crystallization conditions of the template GLIC structure [18] and the presumed open state of the WT receptor [28]. It was recently suggested that GLIC desensitizes on the second time scale [34], and that the GLIC crystal structure may instead represent a desensitized state [33], [34]; however, the pore radius in our WT simulations was sufficient to conduct ions [20] and other studies have shown that similar pore radii were compatible with a conductive state of GLIC [30], [31]. Notably a recent study by Gonzalez-Gutierrez et al. [35] details their infructuous attempts to crystalize ELIC in an open conformation, they conclude that the crystal packing might be more important for energetic conformational equilibrium of LGIC than the presence of agonist or antagonist and mutations favoring the open or close state. The reciprocal should be the same for GLIC, which only introduction of cross-links or non-functional mutations were able to stabilized a locally-closed conformation of GLIC [32]. Given the low deviation of our simulated WT TMD from that of the crystal structure (C_α_ RMSD ∼1.5 Å), our data are consistent with the crystal structure representing an open state. Our group [25] and others [33] also showed that the mutating 14′ position reduced agonist sensitivity, an effect that correlated in this work with constriction of the pore radius and a nonconducting state of the channel. This pore constriction was partially relieved by ethanol binding in the inter-subunit cavity, possibly contributing to the enhanced ethanol potentiation of this mutant. Although our data provide novel insights into ethanol binding to the presumed open state of a pLGIC, alcohols and other modulators may also have relevant interactions with closed, desensitized, or other intermediate states; a complete understanding of allosteric modulation will require modeling of multiple states and the transitions between them. We note that the microsecond timescales of the simulations in this study are still too short to simulate opening, closing, or desensitizing transitions of the channel [28]; instead, our current findings simulate interactions of ethanol with a particular, evidently stable, state of GLIC. The recent determination of GLIC crystal structures in locally closed conformations [32] may lead to valuable new templates for modeling alternative states of this channel; however, the nature of the predominant resting state or states of the channel remain to be determined in detail. Finally, some caution should be exercised when interpreting simulation and experimental results at different pH. These (as other) simulations were performed with constant protonation states that attempt to approximate the pH 4.6 of the GLIC crystal, while experiments have investigated the pH-response of the channel and used EC10 values to perform the actual modulation studies [25]. This is not easily captured in modeling since it is closer to neutral pH where a true constant-pH simulation algorithm would be needed, which is still not in widespread use, in particular not for massively parallel simulations.

### Ethanol potentiation via inter-subunit cavities

The enhanced ethanol potentiation of the F(14′)A mutant corresponded in our simulations to an approximately threefold increase in inter-subunit cavity volume from 96±33 Å^3^ to 283±45 Å^3^. Given that a single ethanol molecule occupies 97 Å^3^, this structural change increased the number of ethanol molecules that could be accommodated by the inter-subunit cavity from ∼1 to ∼3. Accordingly, in our ethanol simulations, F(14′)A increased ethanol binding in the inter-subunit cavity as measured by both occupancy and bulk exchange rate.

Ethanol occupancy increased inter-subunit cavity volume in WT and F(14′)A receptors, possibly associated with the displacement of water by the larger ethanol molecules. This enhancement of inter-subunit cavity volume in the presence of ethanol may provide a structural basis for ethanol potentiation. The inter-subunit cavities are poised to influence channel gating, given their close proximity to the ECD-TMD coupling region [36]; indeed, recent microsecond simulations of GLIC indicated that the shape and volume of these cavities is coupled to channel gating [29]. We previously suggested that ethanol binding in the GlyR might induce swelling of the inter-subunit cavities that prevents the channel from closing or desensitizing [20]. A critical role of inter-subunit cavity volume in ethanol effects may also explain pressure antagonism of ethanol on GlyR function [37]. A correlation between inter-subunit cavity volume and pLGIC potentiation was further supported by the recent crystallization of the eukaryotic pLGIC GluCl [22], in which the partial allosteric agonist ivermectin occupied the inter-subunit interfaces and was associated with enlarged gaps between subunits [22]. Aside from this deviation at the interface, the structure of GluCl aligns closely with that of GLIC, further validating the relevance of this system as a model for eukaryotic pLGIC structure and modulation.

Inter-subunit ethanol binding in F(14′)A was also associated with partial relief from the pore constriction induced by the mutation, which may be directly or indirectly related to changes in inter-subunit cavity volume. Selective occupation of the inter-subunit cavities appeared to be sufficient for this effect, consistent with a negligible contribution of intra-subunit or other binding sites. Based on this observation, ethanol potentiation measured by electrophysiology could reflect compensation for inhibited gating of F(14′)A relative to WT. Indeed, the ∼10% activation level used to test modulation of F(14′)A corresponded to the ∼50% activation level of the WT [25]; thus, if ethanol binding had the sole consequence of restoring F(14′)A to the WT conformation, it would enhance mutant function fivefold. However, our previous electrophysiological studies revealed approximately thirtyfold potentiation of F(14′)A currents by 600 mM ethanol [25], indicating that compensation for reduced gating is not the sole mechanism responsible for ethanol potentiation of these channels. The enhancement of WT GLIC by 600 mM ethanol [25] did not correspond to a change in pore constriction, further implicating an alternative or additional mechanism of potentiation.

In addition to enhancing ethanol potentiation, we previously reported that F(14′)A converted longer-chain alcohols as large as pentanol from inhibitors into potentiators [25]. Modulation by hexanol was biphasic, inhibiting at low concentrations and potentiating at high concentrations; heptanol was weakly inhibiting, with a shallow concentration dependence consistent with simultaneous inhibitory and potentiating interactions [25]. Hexanol (207 Å^3^) and heptanol (236 Å^3^) are too large to bind in the WT inter-subunit cavities (96±33 Å^3^), but would be accommodated by the enlarged cavities in F(14′)A (283±45 Å^3^). Thus, inter-subunit binding may represent a general mechanism for *n*-alcohol potentiation of GLIC F(14′)A.

### Allosteric inhibition via intra-subunit cavities

We previously reported that inhibition of GLIC by *n*-octanol was unaltered by F(14′)A [25], supporting a site of inhibitory action independent of enhanced potentiation in this mutant. Recent co-crystal structures of GLIC bound to the anesthetics desflurane and propofol, both of which inhibited the receptor, supported a mechanism for inhibition via the intra-subunit cavities [23]. Consistent with this model, average intra-subunit cavity volume was unaltered by F(14′)A. Furthermore, we observed equivalent intra-subunit ethanol occupancy in WT and F(14′)A simulations, substantiating this cavity as a binding site for *n*-alcohols and supporting its structural independence from the inter-subunit cavity.

The only intra-subunit difference we observed between WT and F(14′)A was the correlation between ethanol and lipid occupancy: ethanol displaced up to 2 lipid atoms per molecule in the F(14′)A intra-subunit cavity, but displaced only ∼1 lipid atom per molecule in WT. Lipid molecules were resolved in the intra-subunit cavity of the GLIC crystal structure [18], and exhibited higher occupancy in the WT and F(14′)A simulation intra-subunit cavities than in bulk membrane; however, the role of lipids in GLIC function remains unclear. In one possible mechanism, lipids could stabilize the open state by occupying the intra-subunit cavities; displacement of lipids by alcohols or other modulators might disrupt this stabilization and inhibit the receptor. A similar mechanism may underlie the critical role of lipids in stabilizing specific states of the nAChR [9].

Ethanol occupied both inter- and intra-subunit cavities in WT and F(14′)A simulations, suggesting that net modulation might reflect a combination of potentiating and inhibitory binding associated with the inter- and intra-subunit cavities, respectively. By this two-site model, the affinity and efficacy of a given *n*-alcohol in each cavity determine its net functional effect [38]. For example, we previously reported moderate inhibition of F(14′)A by low concentrations and potentiation by high concentrations of hexanol [25]. This relatively hydrophobic alcohol might have greater affinity for the more hydrophobic intra-subunit cavity, and might experience greater accessibility to this cavity via partitioning through the lipid bilayer; thus, at low concentrations, inhibitory effects prevail. At higher concentrations, hexanol might bind with lower affinity to the inter-subunit site, but possibly still have greater efficacy when bound in this site, resulting in a net potentiation of the receptor. Heptanol, which is more hydrophobic than hexanol and almost too large for the inter-subunit cavity, might prefer the intra-subunit cavity even at high concentrations, resulting in net inhibition. Recently, a MD study by Lebard et al. [27] described a negligible affinity of ethanol to the pore and proposed pore blocking as an inhibition site for general anesthetics. However, similar multi-site models of allosteric modulation have been proposed by recent simulation studies of GLIC binding to the volatile anesthetic isoflurane [26], [39].

### GLIC as a model for pLGIC modulation

Most anionic pLGICs, including GlyRs and most GABA_A_Rs, exhibit potentiation by alcohols and anesthetics [1]. Thus, these receptors exhibit a similar profile of modulation to GLIC F(14′)A; and indeed, the position equivalent to F(14′) in anionic pLGICs is generally substituted with a smaller residue [25]. Structure/function studies have identified several residues critical to alcohol and anesthetic potentiation of GlyRs and GABA_A_Rs that map near the GLIC inter-subunit cavities [25]. One early estimate suggested a volume of 189–217 Å^3^ for the GABA_A_R potentiating site [40], between the inter-subunit cavity volumes observed for GLIC WT and F(14′)A. More recently, molecular dynamics simulations of GlyR models based on either GLIC [20] or the low-resolution nAChR template [21] supported ethanol stabilization of the open state via binding in the inter-subunit cavity. Notably, although the dominant modulation exerted by alcohols and anesthetics on anionic pLGICs is positive (potentiating), mutant labeling studies in both GlyRs [41] and GABAaRs [11] also substantiate a negative (inhibitory) modulatory effect exerted via an independent site or sites. Although we cannot rule out contributions of alternative ethanol binding sites, for example in the ECD [42] or ILD [43], to modulation of GlyRs or GABA_A_Rs, the strong correlations of ethanol potentiation with cavity volume, occupancy, and exchange rate in this study highlight an important role for the inter-subunit TMD region. The potent ethanol sensitivity of GLIC F(14′)A in the absence of an ILD suggests this domain is not critical to pLGIC modulation.

Our two-site model of allosteric modulation may be particularly relevant to cationic pLGICs such as nAChRs, which exhibit both potentiation and inhibition by allosteric modulators. Photoaffinity labeling studies localized binding of the potentiator etomidate to an inter-subunit TMD cavity [44], whereas labeling [45] and simulation studies [26] associated inhibitors such as halothane and isoflurane with an intra-subunit cavity. The low-potency inhibitor benzophenone photolabeled both inter- and intra-subunit cavities as well as the channel pore [46]; if these distinct binding sites confer opposing functional effects, their resulting noncompetitive antagonism might underlie the apparent low potency of this agent. Similar to GLIC, nAChRs are potentiated by short-chain alcohols but inhibited by long-chain alcohols [6], and structure/function studies have identified TMD residues that contribute independently to potentiation and inhibition [10]. The conservation of F(14′) in several nAChR subtypes [25] further supports the relevance of WT GLIC as a model for structure, function, and modulation of pLGICs including nAChRs.

## Materials and Methods

### Wild type GLIC and mutated F(14′)A model

The initial GLIC structure was taken from the PDB entry 3EAM [18]. The pdb2gmx program from the GROMACS package [47] was used to add hydrogens according to the residue protonation as defined by Bocquet et al [18]. The mutated F(14′)A GLIC model was built using the backbone-dependent rotamer library SCWRL [48] to mutate phenylalanine 238 (14′ in M2 prime notation) to alanine, and to rebuild side-chains of the mutated residues and the four closest neighbors in the sequence. The ROSETTA refinement program [49] was used to relax the structure, with protonation identical to wild-type.

### MD simulations

Each model was inserted into a pure dioleoylphosphatidylcholine (DOPC) bilayer and overlapping lipid molecules were deleted, keeping 306 DOPC lipids. The two systems were solvated with roughly 34,000 TIP3P water molecules in a hexagonal box. To neutralize the net charge and achieve a physiological ion concentration of ∼100 mM, 61 and 86 water molecules were replaced by Na^+^ and Cl^−^ ions, respectively.

Simulations were performed using GROMACS 4.5.3 [47] with the Amber 03 force field [50] for protein and ions, TIP3P [51] parameters for water, and the Berger force field for DOPC [52]. All bonds were constrained using the LINCS algorithm allowing a time step of 2.5 fs. Particle mesh Ewald electrostatics was used with a 10 Å cutoff for non-bonded interactions and neighborlists updated every 10 steps. Three baths (protein, water and ion, membrane) were coupled to a temperature of 310 K using the Bussi velocity rescaling thermostat with a time constant of τ_T_ = 0.1 ps. The x/y dimensions were scaled isotropically with a Berendsen weak barostat and the z dimension independently to reference pressures of 1 bar, τ_P_ = 1 ps and compressibility of 4.5 · 10^−5^ bar^−1^. The system was minimized for 10,000 steps with steepest descent. It was equilibrated with position restraints of 1000 kJ/mol/nm^2^ on the protein, then for 10 ns with backbone restraints, and finally for 20 ns with only C_α_ restraints. Productions run were performed without any restraints for 1 µs.

Ethanol was added by replacing 1% of the water molecules that were more than 8 Å away from the protein. None were placed inside the protein pore. The system was again subjected to 10,000 steps of minimization. Each system was then used for a 1-µs production run.

We also built a F(14′) GLIC system with one molecule of ethanol docked in each of the five inter-subunit cavities. We used the spontaneously occupied position of ethanol in the inter-subunit cavity in the F(14′)A simulation to place the five ethanol molecules. Among all the ethanol molecules in the F(14′)A simulation, we extracted the coordinates of the ethanol molecule staying the longest in the inter-subunit cavity. Over this portion of the trajectory (∼700 ns), we averaged the ethanol positions and extracted the frame where the ethanol was the closest to the average position. Those coordinates were then imposed on the four other cavities. To keep ethanol molecules in the cavity, distance restraints of 100 kJ/mol/nm^2^ to the initial position were added during the simulation. The system was then subjected to a 500-ns production run. After this period, one ethanol molecule was replaced by a water molecule every 100 ns. These replacements resulted in a system with 5 ethanol molecules between 0 and 500 ns, 4 ethanol molecules between 500 and 600 ns, 3 ethanol molecules between 600 and 700 ns, 2 ethanol molecules between 700 and 800 ns, one ethanol molecule between 800 and 900 ns, and no ethanol between 900 ns and 1.0 µs.

In total, five separate microseconds simulations were performed, and ethanol occupancy analyzed independently for the five different subunits of each protein to increase sampling.

The M2 kink angle was computed within VMD [53] using a custom script, calculating the angle between the two principal axes of inertia of the top and bottom part of M2. The bottom part of M2 was defined by C_α_ of residues 7′–14′ (221–238) and the top part by C_α_ of residues 14′–21′ (238–245).

Average cavity volumes over the course of the simulations were computed in three steps using mdpocket, a module of the Fpocket package [54]. First, mdpocket was used to compute all cavities over the course of the simulation every 5 ns. Second, grids were extracted for intra-subunit and inter-subunit cavities present in at least 20% of the trajectory frames. Onto those 10 cavity grids (5 intra-subunit and 5 inter-subunit), the largest cavity subspace of each type was selected and superimposed on the other 4 cavities of the same type. Third, average volumes were calculated for each cavity within the previously defined grids. All parameters were according to Fpocket defaults, except the volume calculation, for which we used 10,000 Monte Carlo iterations instead of 2,500. Pore radii of the trajectories were computed using the HOLE software [55], extracted each nanosecond and averaged. Average densities were computed using the Volmap plug-in of VMD [53] with a resolution of 1 Å, and averaged over the second half (500 ns) of each trajectory.

## References

[1] Harris RA, Trudell JR, Mihic SJ (2008) Ethanol's molecular targets. Sci Signal 1: re7. 18632551 10.1126/scisignal.128re7 PMC2671803

[2] Howard RJ, Slesinger PA, Davies DL, Das J, Trudell JR, et al (2011) Alcohol-binding sites in distinct brain proteins: the quest for atomic level resolution. Alcohol Clin Exp Res 35: 1561–1573. 21676006 10.1111/j.1530-0277.2011.01502.x PMC3201783

[3] Celentano JJ, Gibbs TT, Farb DH (1988) Ethanol potentiates GABA- and glycine-induced chloride currents in chick spinal cord neurons. Brain Res 455: 377–380. 2900060 10.1016/0006-8993(88)90098-4

[4] Mascia MP, Machu TK, Harris RA (1996) Enhancement of homomeric glycine receptor function by long-chain alcohols and anaesthetics. Br J Pharmacol 119: 1331–1336. 8968539 10.1111/j.1476-5381.1996.tb16042.x PMC1915807

[5] Nakahiro M, Arakawa O, Nishimura T, Narahashi T (1996) Potentiation of GABA-induced Cl- current by a series of n-alcohols disappears at a cutoff point of a longer-chain n-alcohol in rat dorsal root ganglion neurons. Neurosci Lett 205: 127–130. 8907333 10.1016/0304-3940(96)12397-1

[6] Bradley RJ, Sterz R, Peper K (1984) The effects of alcohols and diols at the nicotinic acetylcholine receptor of the neuromuscular junction. Brain Res 295: 101–112. 6608971 10.1016/0006-8993(84)90820-5

[7] Mihic SJ, Harris RA (1996) Inhibition of rho1 receptor GABAergic currents by alcohols and volatile anesthetics. J Pharmacol Exp Ther 277: 411–416. 8613949

[8] Thompson AJ, Lester HA, Lummis SC (2010) The structural basis of function in Cys-loop receptors. Q Rev Biophys 43: 449–499. 20849671 10.1017/S0033583510000168

[9] Baenziger JE, Corringer PJ (2011) 3D structure and allosteric modulation of the transmembrane domain of pentameric ligand-gated ion channels. Neuropharmacology 60: 116–125. 20713066 10.1016/j.neuropharm.2010.08.007

[10] Borghese CM, Henderson LA, Bleck V, Trudell JR, Harris RA (2003) Sites of excitatory and inhibitory actions of alcohols on neuronal alpha2beta4 nicotinic acetylcholine receptors. J Pharmacol Exp Ther 307: 42–52. 14500778 10.1124/jpet.102.053710

[11] Mihic SJ, Ye Q, Wick MJ, Koltchine VV, Krasowski MD, et al (1997) Sites of alcohol and volatile anaesthetic action on GABA(A) and glycine receptors. Nature 389: 385–389. 9311780 10.1038/38738

[12] Hosie AM, Wilkins ME, da Silva HM, Smart TG (2006) Endogenous neurosteroids regulate GABAA receptors through two discrete transmembrane sites. Nature 444: 486–489. 17108970 10.1038/nature05324

[13] Majewska MD, Harrison NL, Schwartz RD, Barker JL, Paul SM (1986) Steroid hormone metabolites are barbiturate-like modulators of the GABA receptor. Science 232: 1004–1007. 2422758 10.1126/science.2422758

[14] Unwin N (2005) Refined structure of the nicotinic acetylcholine receptor at 4A resolution. J Mol Biol 346: 967–989. 15701510 10.1016/j.jmb.2004.12.031

[15] Bertaccini EJ, Trudell JR, Lindahl E (2007) Normal-mode analysis of the glycine alpha1 receptor by three separate methods. J Chem Inf Model 47: 1572–1579. 17602605 10.1021/ci600566j PMC2530920

[16] Miller PS, Smart TG (2010) Binding, activation and modulation of Cys-loop receptors. Trends Pharmacol Sci 31: 161–174. 20096941 10.1016/j.tips.2009.12.005

[17] Hilf RJ, Dutzler R (2008) X-ray structure of a prokaryotic pentameric ligand-gated ion channel. Nature 452: 375–379. 18322461 10.1038/nature06717

[18] Bocquet N, Nury H, Baaden M, Le Poupon C, Changeux JP, et al (2009) X-ray structure of a pentameric ligand-gated ion channel in an apparently open conformation. Nature 457: 111–114. 18987633 10.1038/nature07462

[19] Hilf RJ, Dutzler R (2009) Structure of a potentially open state of a proton-activated pentameric ligand-gated ion channel. Nature 457: 115–118. 18987630 10.1038/nature07461

[20] Murail S, Wallner B, Trudell JR, Bertaccini E, Lindahl E (2011) Microsecond simulations indicate that ethanol binds between subunits and could stabilize an open-state model of a glycine receptor. Biophys J 100: 1642–1650. 21463577 10.1016/j.bpj.2011.02.032 PMC3072665

[21] Cheng MH, Coalson RD, Cascio M (2008) Molecular dynamics simulations of ethanol binding to the transmembrane domain of the glycine receptor: implications for the channel potentiation mechanism. Proteins 71: 972–981. 18004757 10.1002/prot.21784

[22] Hibbs RE, Gouaux E (2011) Principles of activation and permeation in an anion-selective Cys-loop receptor. Nature 474: 54–60. 21572436 10.1038/nature10139 PMC3160419

[23] Nury H, Van Renterghem C, Weng Y, Tran A, Baaden M, et al (2011) X-ray structures of general anaesthetics bound to a pentameric ligand-gated ion channel. Nature 469: 428–431. 21248852 10.1038/nature09647

[24] Weng Y, Yang L, Corringer PJ, Sonner JM (2010) Anesthetic sensitivity of the Gloeobacter violaceus proton-gated ion channel. Anesth Analg 110: 59–63. 19933531 10.1213/ANE.0b013e3181c4bc69 PMC2897236

[25] Howard RJ, Murail S, Ondricek KE, Corringer PJ, Lindahl E, et al (2011) Structural basis for alcohol modulation of a pentameric ligand-gated ion channel. Proc Natl Acad Sci U S A 108: 12149–12154. 21730162 10.1073/pnas.1104480108 PMC3141919

[26] Brannigan G, LeBard DN, Henin J, Eckenhoff RG, Klein ML (2010) Multiple binding sites for the general anesthetic isoflurane identified in the nicotinic acetylcholine receptor transmembrane domain. Proc Natl Acad Sci U S A 107: 14122–14127. 20660787 10.1073/pnas.1008534107 PMC2922517

[27] Lebard DN, Henin J, Eckenhoff RG, Klein ML, Brannigan G (2012) General Anesthetics Predicted to Block the GLIC Pore with Micromolar Affinity. PLoS Comput Biol 8: e1002532. 22693438 10.1371/journal.pcbi.1002532 PMC3364936

[28] Bocquet N, Prado de Carvalho L, Cartaud J, Neyton J, Le Poupon C, et al (2007) A prokaryotic proton-gated ion channel from the nicotinic acetylcholine receptor family. Nature 445: 116–119. 17167423 10.1038/nature05371

[29] Nury H, Poitevin F, Van Renterghem C, Changeux JP, Corringer PJ, et al (2010) One-microsecond molecular dynamics simulation of channel gating in a nicotinic receptor homologue. Proc Natl Acad Sci U S A 107: 6275–6280. 20308576 10.1073/pnas.1001832107 PMC2852019

[30] Fritsch S, Ivanov I, Wang H, Cheng X (2011) Ion selectivity mechanism in a bacterial pentameric ligand-gated ion channel. Biophys J 100: 390–398. 21244835 10.1016/j.bpj.2010.11.077 PMC3021669

[31] Cheng MH, Coalson RD, Tang P (2010) Molecular dynamics and brownian dynamics investigation of ion permeation and anesthetic halothane effects on a proton-gated ion channel. J Am Chem Soc 132: 16442–16449. 20979415 10.1021/ja105001a PMC3071019

[32] Prevost MS, Sauguet L, Nury H, Van Renterghem C, Huon C, et al (2012) A locally closed conformation of a bacterial pentameric proton-gated ion channel. Nat Struct Mol Biol 19: 642–649. 22580559 10.1038/nsmb.2307

[33] Parikh RB, Bali M, Akabas MH (2011) Structure of the M2 transmembrane segment of GLIC, a prokaryotic Cys loop receptor homologue from Gloeobacter violaceus, probed by substituted cysteine accessibility. J Biol Chem 286: 14098–14109. 21362624 10.1074/jbc.M111.221895 PMC3077611

[34] Gonzalez-Gutierrez G, Grosman C (2010) Bridging the gap between structural models of nicotinic receptor superfamily ion channels and their corresponding functional states. J Mol Biol 403: 693–705. 20863833 10.1016/j.jmb.2010.09.026 PMC2966540

[35] Gonzalez-Gutierrez G, Lukk T, Agarwal V, Papke D, Nair SK, et al (2012) Mutations that stabilize the open state of the Erwinia chrisanthemi ligand-gated ion channel fail to change the conformation of the pore domain in crystals. Proc Natl Acad Sci USA 109: 6331–6336. 22474383 10.1073/pnas.1119268109 PMC3341056

[36] Bartos M, Corradi J, Bouzat C (2009) Structural basis of activation of cys-loop receptors: the extracellular-transmembrane interface as a coupling region. Mol Neurobiol 40: 236–252. 19859835 10.1007/s12035-009-8084-x

[37] Perkins DI, Trudell JR, Crawford DK, Alkana RL, Davies DL (2010) Molecular targets and mechanisms for ethanol action in glycine receptors. Pharmacol Ther 127: 53–65. 20399807 10.1016/j.pharmthera.2010.03.003 PMC2891126

[38] Colquhoun D (1998) Binding, gating, affinity and efficacy: the interpretation of structure-activity relationships for agonists and of the effects of mutating receptors. Br J Pharmacol 125: 924–947. 9846630 10.1038/sj.bjp.0702164 PMC1565672

[39] Willenbring D, Liu LT, Mowrey D, Xu Y, Tang P (2011) Isoflurane alters the structure and dynamics of GLIC. Biophys J 101: 1905–1912. 22004744 10.1016/j.bpj.2011.09.026 PMC3192980

[40] Koltchine VV, Finn SE, Jenkins A, Nikolaeva N, Lin A, et al (1999) Agonist gating and isoflurane potentiation in the human gamma-aminobutyric acid type A receptor determined by the volume of a second transmembrane domain residue. Mol Pharmacol 56: 1087–1093. 10531417 10.1124/mol.56.5.1087

[41] Crawford DK, Trudell JR, Bertaccini EJ, Li K, Davies DL, et al (2007) Evidence that ethanol acts on a target in Loop 2 of the extracellular domain of alpha1 glycine receptors. J Neurochem 102: 2097–2109. 17561937 10.1111/j.1471-4159.2007.04680.x

[42] Shen Y, Lindemeyer AK, Gonzalez C, Shao XM, Spigelman I, et al (2012) Dihydromyricetin as a novel anti-alcohol intoxication medication. J Neurosci 32: 390–401. 22219299 10.1523/JNEUROSCI.4639-11.2012 PMC3292407

[43] Yevenes GE, Moraga-Cid G, Romo X, Aguayo LG (2011) Activated G protein alpha s subunits increase the ethanol sensitivity of human glycine receptors. J Pharmacol Exp Ther 339: 386–393. 21821696 10.1124/jpet.111.184408 PMC3199981

[44] Nirthanan S, Garcia G 3rd, Chiara DC, Husain SS, Cohen JB (2008) Identification of binding sites in the nicotinic acetylcholine receptor for TDBzl-etomidate, a photoreactive positive allosteric effector. J Biol Chem 283: 22051–22062. 18524766 10.1074/jbc.M801332200 PMC2494931

[45] Chiara DC, Dangott LJ, Eckenhoff RG, Cohen JB (2003) Identification of nicotinic acetylcholine receptor amino acids photolabeled by the volatile anesthetic halothane. Biochemistry (Mosc) 42: 13457–13467. 10.1021/bi0351561 14621991

[46] Garcia G 3rd, Chiara DC, Nirthanan S, Hamouda AK, Stewart DS, et al (2007) [3H]Benzophenone photolabeling identifies state-dependent changes in nicotinic acetylcholine receptor structure. Biochemistry (Mosc) 46: 10296–10307. 10.1021/bi7008163 17685589

[47] Van Der Spoel D, Lindahl E, Hess B, Groenhof G, Mark AE, et al (2005) GROMACS: fast, flexible, and free. J Comput Chem 26: 1701–1718. 16211538 10.1002/jcc.20291

[48] Canutescu AA, Shelenkov AA, Dunbrack RL Jr (2003) A graph-theory algorithm for rapid protein side-chain prediction. Protein Sci 12: 2001–2014. 12930999 10.1110/ps.03154503 PMC2323997

[49] Rohl CA, Strauss CE, Misura KM, Baker D (2004) Protein structure prediction using Rosetta. Methods Enzymol 383: 66–93. 15063647 10.1016/S0076-6879(04)83004-0

[50] Duan Y, Wu C, Chowdhury S, Lee MC, Xiong G, et al (2003) A point-charge force field for molecular mechanics simulations of proteins based on condensed-phase quantum mechanical calculations. J Comput Chem 24: 1999–2012. 14531054 10.1002/jcc.10349

[51] Jorgensen W, Chandrasekhar J, Madura J, Impey R, Klein M (1983) Comparison of simple potential functions for simulating liquid water. J Chem Phys 79: 926–935.

[52] Berger O, Edholm O, Jahnig F (1997) Molecular dynamics simulations of a fluid bilayer of dipalmitoylphosphatidylcholine at full hydration, constant pressure, and constant temperature. Biophys J 72: 2002–2013. 9129804 10.1016/S0006-3495(97)78845-3 PMC1184396

[53] Humphrey W, Dalke A, Schulten K (1996) VMD: visual molecular dynamics. J Mol Graph 14: 33–38, 27–38. 8744570 10.1016/0263-7855(96)00018-5

[54] Le Guilloux V, Schmidtke P, Tuffery P (2009) Fpocket: an open source platform for ligand pocket detection. BMC Bioinformatics 10: 168. 19486540 10.1186/1471-2105-10-168 PMC2700099

[55] Smart OS, Neduvelil JG, Wang X, Wallace BA, Sansom MS (1996) HOLE: a program for the analysis of the pore dimensions of ion channel structural models. J Mol Graph 14: 354–360, 376. 9195488 10.1016/s0263-7855(97)00009-x

